# Development of Ss-NIE-1 Recombinant Antigen Based Assays for Immunodiagnosis of Strongyloidiasis

**DOI:** 10.1371/journal.pntd.0003694

**Published:** 2015-04-10

**Authors:** Lisa N. Rascoe, Courtney Price, Sun Hee Shin, Isabel McAuliffe, Jeffrey W. Priest, Sukwan Handali

**Affiliations:** 1 Division of Parasitic Diseases and Malaria, Centers for Disease Control and Prevention, Atlanta, Georgia, United States of America; 2 Emory College, Emory University, Atlanta, Georgia, United States of America; 3 Division of Foodborne, Waterborne, and Environmental Diseases, Centers for Disease Controls and Prevention, Atlanta, Georgia, United States of America; Hitit University, TURKEY

## Abstract

*Strongyloides stercoralis* is a widely distributed parasite that infects 30 to 100 million people worldwide. In the United States strongyloidiasis is recognized as an important infection in immigrants and refugees. Public health and commercial reference laboratories need a simple and reliable method for diagnosis of strongyloidiasis to identify and treat cases and to prevent transmission. The recognized laboratory test of choice for diagnosis of strongyloidiasis is detection of disease specific antibodies, most commonly using a crude parasite extract for detection of IgG antibodies. Recently, a luciferase tagged recombinant protein of *S*. *stercoralis*, Ss-NIE-1, has been used in a luciferase immunoprecipitation system (LIPS) to detect IgG and IgG_4_ specific antibodies. To promote wider adoption of immunoassays for strongyloidiasis, we used the Ss-NIE-1 recombinant antigen without the luciferase tag and developed ELISA and fluorescent bead (Luminex) assays to detect *S*. *stercoralis* specific IgG_4_. We evaluated the assays using well-characterized sera from persons with or without presumed strongyloidiasis. The sensitivity and specificity of Ss-NIE-1 IgG_4_ ELISA were 95% and 93%, respectively. For the IgG_4_ Luminex assay, the sensitivity and specificity were 93% and 95%, respectively. Specific IgG4 antibody decreased after treatment in a manner that was similar to the decrease of specific IgG measured in the crude IgG ELISA. The sensitivities of the Ss-NIE-1 IgG_4_ ELISA and Luminex assays were comparable to the crude IgG ELISA but with improved specificities. However, the Ss-NIE-1 based assays are not dependent on native parasite materials and can be performed using widely available laboratory equipment. In conclusion, these newly developed Ss-NIE-1 based immunoassays can be readily adopted by public health and commercial reference laboratories for routine screening and clinical diagnosis of *S*. *stercoralis* infection in refugees and immigrants in the United States.

## Author Summary

Strongyloidiasis is a neglected tropical disease that affects millions worldwide and needs more attention and better diagnostic methods. *Strongyloides stercoralis* can undergo an autoinfection cycle and can cause hyperinfection involving the pulmonary and gastrointestinal systems and disseminated infection in other organs. Although endemic areas are mostly developing countries in tropical and subtropical regions with only sporadic transmission in temperate areas, the disease is a threat to developed world populations through immigrants, refugees, travelers, and military personnel. The disease can have catastrophic effects when a patient is immunocompromised or when an infected organ is transplanted into a vulnerable recipient. Due to the threat to public health, the intricate life cycle of *S*. *stercoralis*, the need to perform multiple follow-up diagnostics to ensure treatment success, and the necessity to rule out multiple co-endemic parasitic infections, it is imperative to develop new diagnostic assays that are simple and efficient while retaining maximal sensitivity and specificity. In this study, we use a well-known recombinant protein, Ss-NIE-1, to optimize assays using both an ELISA format and a multiplex platform to meet these needs.

## Introduction

*Strongyloides stercoralis*, an intestinal nematode that migrates through the skin and lung, is a widely distributed disease that infects 30 to 100 million people worldwide [1]. Unlike other helminthic parasites, *S*. *stercoralis* can complete its entire life cycle within a single human host through autoinfection and can cause an asymptomatic chronic infection that may go undetected for decades in immunocompetent hosts [2, 3]. In the United States, *S*. *stercoralis* causes more deaths than any other soil-transmitted helminth, with mortality rates as high as 87% in cases of hyper-infection in immunocompromised hosts [3].

The standard diagnosis of strongyloidiasis relies on the detection of larvae in the stool [4], but a single stool sample analysis will identify no more than 70% of positive cases [5]. Due to the low sensitivity of the stool assay, immunodiagnosis using a crude antigen-based enzyme-linked immunosorbent assay (ELISA) has been developed as the laboratory test of choice for clinical diagnosis of strongyloidiasis. The Immunoglobulin G (IgG) ELISA utilizes crude extract prepared from L3 *S*. *stercoralis* larvae obtained from infected dogs. Reliance on native parasite materials and the canine infection model are major disadvantages of this test. As a result, a number of recombinant antigen-based ELISAs have recently been developed. Recombinant antigens can be purified easily and can be reproducibly generated in large amounts [6–8]. Antibody detection assays utilizing recombinant protein Ss-NIE-1, a 31-kDa antigen derived from *S*. *stercoralis* L3 parasites [8], have reported sensitivities and specificities of 84–98% and 95–100%, respectively, and are comparable in performance to the crude antigen-based ELISA [6–13].

We have incorporated Ss- NIE-1 into a standard ELISA format assay and into a fluorescent bead format assay (Luminex) to detect *S*. *stercoralis*-specific Immunoglobulin subtype G_4_ (IgG_4_). We have previously used the Luminex system for the simultaneous determination of IgG antibody responses to multiple infections in a single assay run [14–17] and we hope to add the new multiplex bead antibody test to our Neglected Tropical Disease assay panel. We compared the performance of the Ss-NIE-1 recombinant antigen-based ELISA and Luminex bead assays to the published assay performance parameters for the Ss-NIE-1 luciferase immunoprecipitation system (LIPS) based assay [6, 10] and the crude antigen-based IgG ELISA [10]. Because previous research has documented that not all cases of strongyloidiasis are successfully treated with a single course of therapy [18], we also used the fluorescent bead assay to determine if a decrease in antibody was measureable after treatment using a select set of sera.

## Materials and Methods

### Serum Specimens

Although some samples were exhausted during the initial ELISA development and some new samples were added during Luminex assay development, the same sets of sera were used for testing the Ss-NIE-1 ELISA and Ss-NIE-1 Luminex assays and many samples were assayed using both techniques. The sets of human sera used were: (1) samples proven positive for *S*. *stercoralis* based on the presence of larvae in the stool or sputum (ELISA *N* = 258, Luminex *N* = 175); (2) presumed negative samples from U.S. residents with no history of foreign travel (ELISA *N* = 182, Luminex *N* = 207); (3) a convenience panel of samples from patients with various diseases other than *S*. *stercoralis* focusing mainly on worm infections and including 63 sera from proven cases of lymphatic filariasis from Haiti (ELISA *N* = 143, Luminex *N* = 159) [19]; (4) and sera from patients with *S*. *stercoralis* infections, before and after treatment (ELISA *N* = 48, Luminex *N* = 25) [18]. All sera were anonymous and were used in accordance with approved human subjects’ protocols.

### Recombinant Protein Preparation

#### Ss-NIE-1 ELISA antigen

Ss-NIE-1 with a 6x His tag was expressed in *E*. *coli* from a clone in pET30b (kindly provided by T. Nutman, NIAID, NIH, Bethesda, MD) by Genscript (Piscataway, NJ). Expression was analyzed and confirmed by Western Blot using anti- 6xHis antibodies and *S*. *stercoralis* positive serum. The protein was purified in a one-step affinity purification using a Nickel metal affinity column and concentrations were measured with the Bradford protein assay (Bio-Rad Laboratories, Hercules, CA).

#### Ss-NIE-1 Luminex antigen

The *S*. *stercoralis* Ss-NIE-1 antigen coding sequence (GenBank AAB97359) was PCR amplified from a clone in plasmid pET29b (kindly provided by F. Neva, NIAID, NIH, Bethesda, MD) [8] using the following forward and reverse deoxyoligonucleotide primers: 5’-CGC GGA TCC AAT TCG GCA CGA GAT GAA AAT G-3’ and 5’-GCG GAA TTC *TTA* TTG TTT ACG TTG TAA AAC GTT TG-3’, respectively. In these sequences, the restriction sites used for cloning are underlined, and the reverse primer included an in-frame stop codon shown in italics. Previously reported protocols were used for the PCR amplification of the target sequence using AmpliTaq gold DNA polymerase (Perkin-Elmer Cetus, Foster City, CA), for cloning into the BamHI and EcoRI sites of pGEX 4T-2 vector (GE Healthcare, Piscataway, NJ), for expression of the recombinant *Schistosoma* japonicum glutathione *S*-transferase (GST) fusion protein in 2.0 L of *E*. *coli* BL21 cells (Stratagene, La Jolla, CA), and for protein binding to a 10 ml glutathione Sepharose 4B affinity column (GE Healthcare) [20, 21]. Protein was eluted from the column with buffer containing 100 mM Tris at pH 8.0, 500 mM NaCl, and 30 mM reduced glutathione then immediately diluted with 0.33 volumes of buffer containing 10 mM Na_2_HPO_4_ at pH 7.2 and 0.85% NaCl (PBS) with 8 M urea in order to minimize precipitation. The eluate was centrifuged at 26,000 x *g* for 10 min at 4°C, and the supernatant was immediately loaded onto a 50 ml G-25 Sephadex (GE Healthcare) desalting column previously equilibrated with buffer containing 25 mM 2-(*N*-morpholino)-ethanesulfonic acid (MES) at pH 6.0, 200mM NaCl, and 2 M urea. Protein elution was monitored at 280 nm, and three 4 ml fractions were pooled for further purification. Dithiothreitol was added to a final concentration of 1 mM, and the protein was centrifuged as above before loading onto a Mono S HR 5/5 strong cation exchange column (GE Healthcare). Bound protein was eluted at a flow rate of 1 ml/ min with buffer containing 25 mM MES at pH 6.0, 2 M urea, and the following linear NaCl gradients: 0–400 mM NaCl in 4 min, 400–600 mM NaCl in 10 min, and 600–1000 mM in 4 min. A total of 4.2 mg of protein was collected in three 1-ml fractions at approximately 0.44 M NaCl in the gradient profile. Purity was estimated to be >98% by SDS polyacrylamide gel electrophoresis. The recombinant GST/Ss-NIE-1 fusion protein was used in all multiplex assays. Protein concentrations were measured with the BCA microassay (Pierce, Rockford, IL).

### Ss-NIE-1 ELISA Development

The development of an IgG_4_ standard reference curve for the Ss-NIE-1 ELISA was performed as described by Scheel et al [22]. Immunoglobulin G4, human myeloma plasma was purchased and stored frozen in 20 mM phosphate, pH 7.4, with 150 mM NaCl and 0.05% Sodium azide (NaN_3_) (Athens Research & Technology, Athens, GA). IgG_4_ was diluted into antigen sensitizing buffer (ASB) (0.05 M Tris/HCl, pH 8.0 + 1 M KCl + 2 mM EDTA) to create standard curve points. IgG_4_ concentrations were chosen based on previous experiments in standard curve development and adjusted to produce the highest OD value, ~ 2.0.

Checkerboard titrations for antigen concentration, serum dilution, conjugate dilution, and substrate 3, 3’, 5, 5’-Tetramethylbenzidine (TMB) time were carried out on Immulon 2HB Microwell plate (Thermo Scientific, Cat. Number 6506). For optimization of the Ss-NIE-1 ELISA, optimal conditions were chosen based on the signal to noise ratio between defined strong *S*. *stercoralis* positive and normal human serum samples. The optimized Ss-NIE-1 ELISA steps are as follows: the micro-well plate was sensitized with 100 μL/well of Ss-NIE-1 antigen at a concentration of 0.3 μg/mL in antigen sensitizing buffer (0.05 M Tris/HCl, pH 8.0 + 1 M KCl + 2 mM EDTA) for 2 hours at room temperature on a plate shaker. Following antigen sensitization, the plate was washed 4 times with PBS/0.3% Tween. The plate was blocked for 30 minutes with 100 μL/well of 10 mM Nickel Chloride (Aldrich, Cat. Number 339350) in PBS/0.3% Tween/5% Instant Nonfat Dry Milk (Nestle, Glendale, CA), and then washed as before. StabilCoat Immunoassay Stabilizer (SurModics, East Prairie, MN) was then added 100 μL to each well and incubated for 30 minutes on a plate shaker at room temperature. After discarding the blocking solution, the plate was dried for 4 hours at 30°C in a vacuum oven chamber. The sensitized and blocked plate was stored at 4°C in sealed aluminum foil with desiccator.

### Ss-NIE-1 ELISA Protocol

Human serum samples were tested in 100 μL/well at 1:50 dilution in PBS/0.3% Tween/5% Instant Nonfat Dry Milk. Following 30 minutes incubation at room temperature on a plate shaker (speed ~ 800 rpm), the plate was washed 4 times with PBS/0.3% Tween. Proper conjugate concentration of mouse anti-human IgG4 (clone HP6025), affinity purified, horseradish peroxidase labeled (Southern Biotech, Birmingham, AL; Cat. Number 9200–05) was added to each well at 100 μL/well at 1:1,000 dilution in PBS/0.3% Tween and incubated 30 minutes at room temperature on a plate shaker with the plate being washed 4 times following incubation with PBS/0.3% Tween. The substrate used was SureBlue, 3, 3’, 5, 5’-Tetramethylbenzidine (TMB) Microwell Peroxidase Substrate (KPL, Gaithersburg, Maryland). We used 100 μL/well of TMB to develop the plate for 5 minutes, and the reaction was stopped by adding 100 μL 1 N H_2_SO_4_ Analyzed ACS Reagent (J.T. Baker, Phillipsburg, NJ). The signal was read at A_450nm_ using a VersaMax Kinetic ELISA Microplate Reader with SoftMax Pro v5.4 Software (Molecular Devices Corporation, Palo Alto, CA).

### Ss-NIE-1 Luminex Assay Development

#### Protein coupling to MagPlex magnetic beads

Coupling of protein to MagPlex Magnetic Microspheres (Luminex, Austin, TX) was carried out using EDC-Sulfo NHS protocol [23, 24]. Briefly, beads were washed and activated in buffer containing 50mM MES, pH 5, 0.85% NaCl, and 0.05% Tween-20. After 40 minutes of incubation using end-over-end mixing in the dark with Sulfo-NHS and EDC, beads were washed 2 times with MES buffered saline. The activated beads were then transferred to a new tube and washed once more. Beads were resuspended in the MES buffer without Tween-20 and 0.3 μg of GST-Ss-NIE-1/1.25 x 10E6 beads were added. The total volume of reaction was brought to 500 μL with MES buffer without Tween-20. The coupling was performed for 3 hours in the dark at room temperature by end-over-end mixing. Beads were blocked with blocking buffer (PBS + 1% BSA + 0.05% sodium azide (NaN_3_), pH 7.4) for 30 minutes. The coupled beads were stored at 4°C in PBS + 1% BSA + 0.05% NaN_3_ + 0.05% Tween-20 + PMSF (1:500), Pepstatin (1:1000), Leupeptin (1:1000) until used. The concentration of the beads was determined by viewing them in a hemocytometer using a 20X objective.

#### Luminex immunoassay

Fifty μL of the working MagPlex microsphere mixture (50 beads/μL in PBS/0.3% Tween-20/5%—Instant Nonfat Dry Milk) and 50 μL of diluted sera (1:100 in PBS/0.3% Tween-20/5% Instant Nonfat Dry Milk) were added into each well of Costar 96-well black, round-bottom plate (Fisher Scientific, Cat.# 3792). After 30 minutes incubation at room temperature with shaking at speed 6 (~800 rpm), the beads were washed using the Biotek Magnetic Washer ELx50 (2 minutes magnetic separation followed by 2 cycles of dispensing 100 μL of PBS/Tween-20 0.3% and a 40 second soak before aspiration). The complex of antibody and coupled beads was detected using 50 μL of biotinylated mouse anti-human IgG_4_ (clone HP6025, affinity purified, Southern Biotech, Birmingham, AL, Cat. Number 9200–08) diluted 1:200 in PBS-1% BSA, 0.05% NaN_3._ After 30 minute incubation, the beads were washed as previously described. As a detector, 50 μL/well of *R*- phycoerythrin-labeled streptavidin conjugate (Invitrogen, Cat. # S866) at a 1:250 dilution in PBS-1% BSA, 0.05% NaN_3_ was added to the well and incubated for 30 minutes. After washing, the beads were resuspended using 100 μL/well of PBS-1% BSA, 0.05% NaN_3_. The mean fluorescence intensity from each well was determined by using BioPlex manager software, version 6.02 (BioRad) and a Luminex 100 platform.

### Data Analysis

Data were tabulated and analyzed using Microsoft Excel. Determination of the cut-off value and assay performance was calculated using SAS version 9.0. The concentration of Ss-NIE-1 ELISA was measured in in ng/mL; Luminex results are reported as mean fluorescence intensity minus background blank (MFI). The J-index, a single measurement of assay performance, was calculated as described previously ([25, 26].

## Results

### *S*. *stercoralis* NIE-1 IgG_4_ ELISA

The recombinant Ss-NIE-1-His protein expressed at Genscript was successfully used to develop an ELISA (Table 1). Using a cutoff value of 0.80 ng IgG_4_/mL, the assay correctly identified 245 of 258 parasitologically confirmed strongyloidiasis cases for a sensitivity of 95% (Table 1). The overall specificity of the ELISA was determined to be 93% using a panel of non-endemic US controls and sera from patients with other (mainly parasitic worm) diseases (Tables 1 and 2). Among the 142 donors with defined diseases or parasitic infections shown in Table 2, the specificity was only 86%, but only the two trichuriasis patients were 100% cross-reactive. Although the numbers of samples were also quite small, cross reactivities of ≥ 33% were observed among sera from echinococcosis, gnathostomiasis, and hookworm patients. Table 2 shows the cross-reactivity of the various presumed negative sera. Given the likelihood of polyparasitism among many of these serum donors (i.e., 63 lymphatic filariasis patients from *S*. *stercoralis*-endemic Haiti), the 99% specificity observed among US negative controls may be a reasonable upper bound to the value. The J-index, a single measure of assay performance, was 0.88.

**Table 1:** Performance of SS-NIE-1 ELISA and Luminex.

Assay Characteristics	SS-NIE-1 ELISA	95% CI *	Ss-NIE-1 Luminex	95% CI
Cut-off point	0.8 ng IgG_4_/ mL		8 MFI units	
J-index	0.88		0.88	
Sensitivity: *N* Positive/ *N* Tested (%)	245/258 (95)	92–97	162/175 (93)	88–96
Specificity: *N* Negative/ *N* Tested (%)	303/325 (93)	90–96	349/366 (95)	93–97

Note: * CI = confidence interval

**Table 2:** Cross-reactivity of SS-NIE-1 ELISA and Luminex.

**SS-NIE-1 ELISA**				**SS-NIE-1 Luminex**		
Cross reactivity (%)	No. of positives	No. of sera tested	Conditions represented by sera	No. of sera tested	No. of positives	Cross reactivity (%)
**1**	**2**	**182**	**U.S. Negatives**	**207**	**3**	**1**
**NT**	NT	NT	**Amebiasis**	2	1	50
**7**	1	14	**Ascariasis**	8	1	13
**0**	0	1	**Baylisascariasis**	1	0	0
**50**	1	2	**Cancer**	2	0	0
**0**	0	2	**Cysticercosis**	4	0	0
**NT**	NT	NT	**Dirofilariasis**	1	0	0
**50**	1	2	**Echinococcosis**	5	0	0
**0**	0	2	**Eosinophilic myalgia**	1	0	0
**10**	6	63	**Filiariasis**	63	6	10
**50**	2	4	**Gnathostomiasis**	NT	NT	NT
**33**	2	6	**Hookworm *(N. americanus)***	14	2	14
**NT**	NT	NT	**Hymenolepiaisis**	3	1	33
**NT**	NT	NT	**Paragonimiasis**	6	0	0
**0**	0	4	**Schistosomiasis**	5	0	0
**NT**	NT	NT	**Taeniasis**	4	0	0
**12**	4	33	**Toxocariasis**	28	1	4
**0**	0	4	**Toxoplasmosis**	3	0	0
**25**	1	4	**Trichinellosis**	4	1	25
**100**	2	2	**Trichuriasis**	4	1	25
**NT**	NT	NT	**Tuberculosis**	1	0	0

NT = Not tested

Positive sera with low and medium level reactivity were used to measure inter-assay variation as previously described in the Materials and Methods. The inter-assay coefficient of variation was determined to be 22% for the low positive control serum and 10% for the medium positive control serum.

### Fluorescent Bead Ss- NIE-1 IgG_4_ Assay

The His tagged Ss-NIE-1 recombinant protein could not be coupled to magnetic beads. Thus, we proceeded using a GST-tagged Ss-NIE-1 protein and successfully coupled the Ss-NIE-1 protein to the MagPlex microspheres. The intra- and inter-assay coefficients of variation were determined to be 4.2% and 13.9%, respectively, for average values of 641 MFI and 585. Using a cutoff value of 8 MFI, the sensitivity and specificity of the IgG_4_ Luminex assay were 93% and 95% (Table 1), respectively. As with the ELISA described above, the specificity among US negative controls was much higher (99%) than among donors with defined diseases or parasitic infections (91%) (Table 2). High reactivities (≥33%) were only observed among sera from the two amebiasis and three hymenolepiasis donors. The J-index was identical to that of the ELISA at 0.88.

### Post Treatment Analysis

Antibody longevity in subjects infected with strongyloidiasis following treatment with thiabendazole [18] can be seen in Table 3. Peak and median antibody responses decreased over time using both assay formats, but the antibody levels remained above the cut-off point for most of the subjects even 18 months post treatment. Using the Kobayashi criteria of cure, which considers a patient to be cured if the ratio of serological results post-treatment compared to pre-treatment is less than 0.6, 70% of the subjects would be reported as cured 3–6 months following treatment [27].

**Table 3:** Change in antibody response following treatment.

	At baseline		< 3 MONTHS		3–6 MONTHS		7–9 MONTHS		10–18 MONTHS		> 18 MONTHS	
	ELISA†	Luminex†	ELISA	Luminex	ELISA	Luminex	ELISA	Luminex	ELISA	Luminex	ELISA	Luminex
**No. tested**	48	25	2	1	26	11	21	12	22	12	9	7
**Range**	0–255	11–20321	0.5–9.7	480	0–89	3–9483	0.5–77	8–7447	0–33.3	15–6440	0–16.5	20–3324
**Mean**	28.8	3247	5.1	480	14.4	1663	16.3	1622	7.5	1293.3	3.7	1118
**Median**	13.8	596	5.1	480	5.9	448	4.6	241	3.5	168	1.8	81
Ratio < 0.6 (%) *			50	0	70	82	68	73	88	83	71	71
**Ratio Range**			0.3–2.3	0.7–0.7	0.1–1.4	0.2–0.9	0.1–2.2	0.1–0.8	0–3.3	0.1–1.4	0–2.4	0.1–2.3

Note:

† For SS-NIE-1 ELISA, units are expressed in ng/mL and for SS-NIE-1 Luminex, the unit is a ratio of mean fluorescent intensity minus the background of the test/control.

*Follow-up serologic result divided by the initial result

## Discussion

Strongyloidiasis is an increasingly important health problem in the US among immigrants and refugees. Patients with occult strongyloidiasis are at risk of disease if they become immunosuppressed, and organ donors with unrecognized *S*. *stercoralis* infection pose a risk to recipients if their infected organs are transplanted [2]. Identification of the parasite in stool specimens is insensitive, and, because parasitological examination requires collection of multiple stool specimens over 3 days, serological testing would be preferred if available. We elected to develop novel immunodiagnostic assays to meet this need. We employed a well described recombinant protein with proven performance as an immunodiagnostic antigen, the Ss-NIE-1 protein [6–8]. Based on the potential importance of IgG_4_ antibody responses in filarial infections, we decided to develop methods that detect *S*. *stercoralis*-specific IgG_4_ antibodies. The Ss-NIE-1 IgG_4_ Luminex bead assay achieved a sensitivity and a specificity comparable to those reported for other strongyloidiasis assays such as the crude antigen ELISA, and 26-kDA ELISA, and Ss-NIE-1 ELISAs and the Ss-NIE-1 LIPS [6, 7, 9, 10, 12, 18]. Compared to the CDC *S*. *stercoralis* crude antigen ELISA, the Ss-NIE-1 IgG_4_ ELISA and the Ss-NIE-1 IgG_4_ Luminex assay achieved similar sensitivity without compromising specificity. The possible factors contributing to improved specificity could be the use of recombinant antigen, assay optimization, or detection of IgG_4_ versus IgG. During Ss-NIE-1 ELISA optimization, we found that non-specific antibody binding in the normal human sera could be decreased by adding a pre-blocking step with 10 mM nickel chloride in PBS/0.3% Tween/5% milk. The decrease in background noise allows the assay to have a higher specificity (93%).

ELISA based tests can only be used to detect antibody responses against one antigen of interest at a time. Because the differential diagnosis of *S*. *stercoralis* often includes multiple helminths, a submitted serum sample must frequently be tested using several parasite-specific ELISAs to determine the possible cause(s) of infection. xMAP Luminex technology offers an assay platform that can simultaneously detect antibodies to multiple diseases/infections, and multiplex bead-based antibody assays are generally as sensitive as conventional ELISA, have a wide dynamic range, and are highly reproducible from assay to assay [28]. For these reasons, we elected to transfer the Ss-NIE-1 assay to the Luminex platform. In our hands, the Ss-NIE-1 IgG_4_ Luminex bead assay was slightly less sensitive and slightly more specific than the ELISA, but had comparable overall performance as measured by the J-index.

With the exception of toxocariasis and lymphatic filariasis, our cross-reactivity data must be interpreted with some caution due to the small numbers of samples available for testing. When sera from echinococcosis, hookworm, and trichuriasis patients were tested with Ss-NIE-1 by ELISA, 33% or more of the sera reacted, some quite strongly. A portion of this reactivity likely resulted from ELISA-specific background as many of these same sera did not react with the Ss-NIE-1 antigen in our newly developed Luminex bead assay. Bisoffi et al. [9] found no ELISA or LIPS assay cross-reactions to Ss-NIE-1 among their *Echinococcus-* or hookworm-infected donors. Although true cross-reactivity between these parasites may exist, polyparasitism cannot be ruled out in the remaining Luminex-positive subjects. An analysis of Ss-NIE-1 LIPS results from a hookworm/ *Ascaris lumbricoides*/ *H*. *nana*/ *S*. *stercoralis* co-endemic region of Argentina failed to demonstrate an association between infection with other parasites and an antibody response to the Ss-NIE-1 antigen [10].

The Ss-NIE-1 ELISA and Luminex had 10% cross-reactivity against the subjects with lymphatic filariasis. Again, this problem could be a true cross-reactivity or could also be explained by polyparasitism with soil-transmitted helminthes in Haiti. Unfortunately, we have no data about the presence of other parasitic infections in the individuals from whom these serum samples were obtained [29]. Norsyahida [11] reported that although the use of IgG_4_ conjugate did decrease the cross-reactivity to filariasis compared to the total IgG responses, some cross-reactivity was found with the IgG_4_ based assay. However, the Norsyahida group did not use a recombinant antigen [8], and the observed cross-reactivity could be due to the use of crude extract antigen in their assay. Of note, the group mentioned the importance of testing for filariasis in subjects with strongyloidiasis. Such testing could most easily be accomplished on a multiplexing platform such as Luminex that can detect antibodies against filariasis and strongyloidiasis simultaneously.

Decreases in antibody titers post-treatment were observed (as suggested by Satoh [30]) and the percentage of patients who met the serologic definition of cure (≥40% decline in antibody response compared to pre-treatment) were consistent with the previous reports [18]. The only significant difference was that the CDC *S*. *stercoralis* crude ELISA results showed that 92%of subjects at >9–18 months had been cured of *S*. *stercoralis* infection, but only 71% could be considered as cured based on the SS-NIE-1 ELISA and Luminex assay. We have no definitive explanation for these observed differences, except the fact that the crude ELISA uses a complex antigen versus a single antigen which was used in our studies.

Overall, excellent assays for detecting *S*. *stercoralis* specific antibodies have been developed. Because these assays use recombinant proteins, negating the need for native parasite materials these assays can be adopted for use in public health laboratories for refugee screening or in commercial laboratories for diagnosis of clinical strongyloidiasis and for screening possible transplant donors with occult disease. As both ELISA and Luminex based assays performed similarly, studies in low infrastructure, endemic setting could use the ELISA format. Although polyparasitism is a potential problem, with a strong specificity of 93%, cross-reactivity should not be an issue. For a country-wide study to determine the prevalence of strongyloidiasis, a multiplexing capability of Luminex will be more cost efficient.
